# Automation in urinalysis: evaluation of three urine test strip analysers

**DOI:** 10.1155/S1463924688000239

**Published:** 1988

**Authors:** Pierangelo Bonini, Lucilla C. Sanguini, Laura Grossi, Ferruccio Ceriotti, Michelangelo Murone

**Affiliations:** Istituto Scientifico H. S. Raffaele Laboratorio Analisi Via Olgettina 60 Milano 20132 Italy

## Abstract

A clinical laboratory evaluation was conducted on the Clinitek Auto 2000, the Super Aution Analyzer and the Urotron RL9 for the determination of glucose, protein, pH, blood, ketone-bodies and bilirubin.

Precision of the systems was tested using three commercial control urine materials, and reported as the percentage of times the instrument repeats a certain value. Good repeatability was obtained with all the instruments.

Accuracy of the systems was evaluated by comparison with quantitative procedures, and to check agreement between methods yielding semi-quantitative and quantitative results, ranges of acceptability were defined, based on the criteria reported in a previous paper [2]. It was then found that 87.5 to 98.9% of results from the Urotron RL9 and the Clinitek Auto 2000 were acceptable. With the Super Aution Analyzer the level of agreement was apparently lower because of the higher number of concentration steps used by this instrument.


Journal of Automatic Chemistry, Vol. 10, No. 3 (July–September 1988), pp. 121–129

### Automation in urinalysis: evaluation of three urine test strip analysers

**Pierangelo Bonini, Lucilla C. Sanguini, Laura Grossi, Ferruccio Ceriotti and Michelangelo Murone**

*Istituto Scientifico H. S. Raffaele, Laboratorio Analisi, Via Olgettina 60, 20132 Milano, Italy*

*A clinical laboratory evaluation was conducted on the Clinitek Auto 2000, the Super Aution Analyzer and the Urotron RL9 for the determination of glucose, protein, pH, blood, ketone-bodies and bilirubin.*

*Precision of the systems was tested using three commercial control urine materials, and reported as the percentage of times the instrument repeats a certain value. Good repeatability was obtained with all the instruments.*

*Accuracy of the systems was evaluated by comparison with quantitative procedures, and to check agreement between methods yielding semi-quantitative and quantitative results, ranges of acceptability were defined, based on the criteria reported in a previous paper [2]. It was then found that 87·5 to 98·9% of results from the Urotron RL9 and the Clinitek Auto 2000 were acceptable. With the Super Aution Analyzer the level of agreement was apparently lower because of the higher number of concentration steps used by this instrument.*

### Introduction

Urinalysis is now a very commonly performed, routine procedure and with the introduction of 'dipsticks' detection and measurement has become rapid and practical. More recently, the introduction of automatic analysers based on reflectance photometry has overcome the difficulty of subjective visual interpretation and allowed the achievement of better standardization.

The aim of this study was to investigate the performance of the most recently developed instruments: Clinitek Auto 2000 (Ames Co., Division of Miles Laboratory, Cavenago Brianza, Milano, Italy), Super Aution Analyzer SA4220 (Kyto Daiichi, Kogaku, represented in Italy by A. Menarini, Firenze) and Urotron RL9 (Boehringer Biochemia Robin, Milano, Italy).

The use of international guidelines for the evaluation of clinical chemistry analysers, designed for instruments providing quantitative results, gives rise to some difficulties when applied to instruments producing semiquantitative values [1 and 2]. Nevertheless, an endeavour was made to follow standardized protocols proposed for the evaluation of analysers in clinical chemistry [3 and 4] and their practicability was tested for analytical procedures leading to semiquantitative results.

In the evaluation, findings for urinary pH, protein, glucose and blood were compared with those from chosen quantitative methods. For bilirubin and ketone bodies appropriate quantitative methods were not easily available, so only recovery experiments were performed. Urobilinogen was not studied because there was no satisfactory reference material available nor was there a valid and practical comparison method. Leukocytes and nitrite were not taken into account.

### Materials and methods

### *Instruments*

Characteristics of the three urine-analysers are given in table 1. The directions of the manufacturer were used throughout. The Clinitek Auto 2000 and Super Aution Analyzer were calibrated every day. Urotron RL9 was calibrated every two weeks as recommended by the manufacturer, but the entire system was checked daily using an appropriate control strip (supplied by Boehringer).

### *Reagent strips*

The Urotron RL9, Combur9Test RL strips have to be dipped manually into each urine sample following an optical and acoustic signal. The strips are then read automatically within 1 min. The period of immersion of test strips in the specimen is standardized at 2 s.

The Super Aution Analyzer employs a robot arm to provide complete automated operation for the entire procedure, from picking up the test strip to measurement.

Reagent cassettes are used with the Clinitek Auto 2000 and contain a roll of plastic film with 400 separate reagent areas attached. These are impregnated with chemicals for the determination of one type of analyte. Clinitek Auto 2000 performs urine-analysis by automatically aspirating and dispensing 100–200 µl of the urine sample on each test pad.

### *Patients' specimens*

Fresh urine samples submitted to the laboratory were selected for the study and stored at 4 °C for a maximum of 4 h until analysed. To avoid interference no preservative was added to the samples.

It is well known that ascorbic acid may interfere with some tests on urine chemistry strips [5] and for this reason only fresh urine samples were selected after a negative ascorbic acid test by Rapignost Total Screen R (Istituto Behring, Scoppito, Aquila).

For the recovery of experiments, normal urine samples were collected from laboratory personnel. The specimens were negative for glucose, protein, ketones, bilirubin,

*Table 1. Characteristics of the three urine-analysers.*

| | Clinitek Auto 2000 | Super Aution Analyzer | Urotron RL9 |
|---|---|---|---|
| Reagent strips | Reagent cassettes | Uriflet 8A | Combur9Test RL |
| Optical configuration | Interference filters and fibre optics | Dual wavelength reflectance measurement by means of spherical integrator | 11 channel reflectance with 11 LEDs and 11 phototransistors |
| Wavelengths (nm) | 510, 530, 550, 570, 630, 650, 670, 690 | 550, 620, 720 | 557, 608, 634, 665 |
| Compensation field | // | Present | Present |
| Strip transport mechanism | Moving pins advance strips under two read-heads. Mechanism transports many strips at one time | A robot arm provides automatic positioning of test strips under measuring head and removes test strips after reading | Paper is used as the transport medium for the moist test strips |
| Sample throughput | 120/h | 250/h | 150–300/h |

haemoglobin and urobilinogen as shown by the three urine-analysers. Relative density and pH were in the normal ranges.

*Urine control materials*

Lyphochek control urine II (Bio-Rad, Segrate, Milano) and Kova-Trol I and II (Boehringer Biochemia Robin, Milano) were used to evaluate the precision of the systems.

*Reagents*

Crystalline bilirubin (n.4126) was purchased from Sigma Chemical Co. (St. Louis, Missouri). Aqueous acetoacetic acid solution was supplied by Miles Italiana (Cavenago Brianza, Milano) and lyophilized human albumin, used as standard for total protein determination, was obtained from Boehringer, Mannheim, GmbH. All other reagents were of pure analytical grade.

*Comparison methods*

Comparison studies with patients' urine samples were carried out for glucose using a hexokinase/G6PDH method [6] on a Hitachi 737 analyser and protein using Coomassie brilliant blue [7] (manual determination); pH was measured on a Radiometer PHM 84 instrument. Each sample was analysed once on each automatic system and with a quantitative method for comparison. The time between dipstick analysis and quantitative analysis never exceeded 2 h.

*Recovery experiments*

For erythrocyte detection and measurement, 0·1 ml of an EDTA treated whole blood sample containing $5{\cdot}3 \times 10^6$ erythrocytes/μl was added to 99·9 ml of 0·9% NaCl solution. Different aliquots of this stock solution were diluted to 12 ml with normal urine (free of blood) to give final concentrations between 5 and 600 erythrocytes/μl. For reference a cell count was performed on an uncentrifuged urine using a Neubauer counting chamber.

For ketone-bodies and bilirubin artificially supplemented urines were prepared by adding pure substances. Results obtained from the automatic urine-analysers were compared with the theoretical concentrations.

### Results and discussion

*Imprecision*

Ten determinations were performed with each urine control material on five consecutive days. The results are summarized in tables 2–4.

Standard deviations and coefficients of variation cannot be calculated for test strip analysers operating in discrete mode, so in the tables the percentage of times the instrument repeats a certain value is indicated. Good repeatability is reported as 100% when the same value is always obtained. A degree of imprecision may depend on analyte concentration being at the border between two concentration ranges.

For this reason, with these instruments, a variation of one step lower or higher than the expected one [8] is generally accepted to be a reasonably good precision.

Anomalies were found using experimental control material, particularly with the Super Aution Analyzer and with the Urotron RL9 for bilirubin in Kova-Trol I (see table 4). This variability can be largely attributed to the characteristics of the testing materials. Good repeatability was observed with patients' samples.

*Method comparison*

Statistical evaluation of comparisons between methods yielding semiquantitative and quantitative results is difficult and here quantitative results have been grouped in 'concentration ranges' centred around a semiquantitative set point, indicated by the instruments.

In the absence of a precise indication by the manufacturer, the borderline values of a concentration range, were set exactly half-way between two adjacent set points [9 and 10]. As in a previous paper [2] agreement between the various analysers and a quantitative comparison method was considered good if the value obtained with the comparison method did differ from the concentration range of a set point, plus or minus 50% of the adjacent ranges. The results of the comparison experiments are reported in figures 1–4; the solid lines indicate the acceptable ranges.

For the Clinitek Auto 2000, results are in good agreement with those of the comparison methods for protein and pH, only 1·1% and 6·2% of specimens respectively gave results outside the acceptable range. For glucose, the agreement was complete (figure 2[*a*]).

With the Urotron RL9 good results were determined for glucose – no outlier (figure 2[*b*]) and proteins – 4% out of acceptable range (figure 1[*b*]). Results from pH (figure 3[*b*]) were poor (12·5% not acceptable), despite the concentration ranges being double those of the other instruments.

The results from the Super Aution Analyzer need to be carefully evaluated; higher percentages of values outside the acceptable range (37·5% for glucose, 50·5% for protein and 27·1% for pH) are mainly due to the greater number of concentration intervals which is a feature of this instrument. Since precision is similar to the other two instruments, it is suggested that the number of intervals should be made the same. With the instrument having a higher number of intervals (and, consequently, very small ranges of concentration), the increased number of results outside the acceptable intervals is understandable.

Figure 4 shows the results obtained when different concentrations of erythrocytes were added to normal urines. With the Urotron RL9, 92·2% of results agreed with the comparison method. With the Super Aution Analyzer, 41·2% of results were underestimated. This

*Table 2. Within-day and between-day precision. (Clinitek Auto 2000.)*

| | | | | Within day | | | | | Between day |
|---|---|---|---|---|---|---|---|---|---|
| | | Ref. | ** | 1 $N=10$ | 2 $N=10$ | 3 $N=10$ | 4 $N=10$ | 5 $N=10$ | $N=5$ |
| | pH | 6·5 | 6·5 | 100 | 100 | 100 | 100 | 100 | 100 |
| | Protein | 92 | 100 | 10 | 0 | 0 | 0 | 0 | 2 |
| | (mg/dl) | | 300 | 90 | 100 | 100 | 100 | 100 | 98 |
| | Glucose | 243 | 250 | 80 | 100 | 100 | 20 | 100 | 80 |
| Lyphochek II | (mg/dl) | | 500 | 20 | 0 | 0 | 80 | 0 | 20 |
| | Blood | | Mod | 100 | 100 | 100 | 100 | 100 | 100 |
| | Bilirubin | | Neg | 100 | 100 | 100 | 100 | 100 | 100 |
| | Ketones | | Neg | 100 | 100 | 100 | 100 | 100 | 100 |
| | (mg/dl) | | | | | | | | |
| | pH | 8·5 | 9·0 | 100 | 100 | 100 | 100 | 100 | 100 |
| | Protein | 224 | 300 | 60 | 70 | 100 | 100 | 50 | 76 |
| | (mg/dl) | | 2000 | 40 | 30 | 0 | 0 | 50 | 24 |
| | Glucose | 1204 | 1000 | 0 | 60 | 80 | 30 | 60 | 46 |
| Kova-Trol I | (mg/dl) | | 2000 | 100 | 40 | 20 | 70 | 40 | 54 |
| | Blood | | Large | 100 | 100 | 100 | 100 | 100 | 100 |
| | Bilirubin | | Large | 100 | 100 | 100 | 100 | 100 | 100 |
| | Ketones | | 15 | 100 | 100 | 100 | 100 | 100 | 100 |
| | (mg/dl) | | | | | | | | |
| | pH | 7·5 | 8·5 | 100 | 80 | 80 | 100 | 100 | 92 |
| | | | 9·0 | 0 | 20 | 20 | 0 | 0 | 8 |
| | Protein | 38 | 30 | 100 | 100 | 100 | 100 | 100 | 100 |
| | (mg/dl) | | | | | | | | |
| Kova-Trol II | Glucose | 183 | 250 | 100 | 100 | 70 | 0 | 60 | 66 |
| | (mg/dl) | | 500 | 0 | 0 | 30 | 100 | 40 | 34 |
| | Blood | | Small | 100 | 100 | 100 | 0 | 100 | 80 |
| | | | Trace | 0 | 0 | 0 | 100 | 0 | 20 |
| | Bilirubin | | Neg | 0 | 0 | 0 | 100 | 0 | 20 |
| | | | Small | 100 | 100 | 100 | 0 | 100 | 80 |
| | Ketones | | 5 | 100 | 0 | 0 | 0 | 0 | 20 |
| | (mg/dl) | | 15 | 0 | 100 | 70 | 100 | 100 | 74 |
| | | | 40 | 0 | 0 | 30 | 0 | 0 | 6 |

** Instrument display levels.

*Table 3. Within-day and between-day precision. (Super Aution Analyzer.)*

| | | | | Within day | | | | | Between day |
|---|---|---|---|---|---|---|---|---|---|
| | | Ref. | ** | 1 $N=10$ | 2 $N=10$ | 3 $N=10$ | 4 $N=10$ | 5 $N=10$ | $N=5$ |
| | pH | 6·5 | 6·5 | 100 | 20 | 60 | 100 | 100 | 76 |
| | | | 7·0 | 0 | 80 | 40 | 0 | 0 | 24 |
| | Protein | | 200 | 40 | 0 | 10 | 50 | 0 | 20 |
| | (mg/dl) | 92 | 250 | 40 | 10 | 30 | 40 | 30 | 30 |
| | | | 300 | 10 | 60 | 20 | 10 | 40 | 28 |
| | | | 400 | 10 | 30 | 40 | 0 | 30 | 22 |
| | Glucose | | 200 | 0 | 20 | 0 | 0 | 0 | 4 |
| Lyphochek II | (mg/dl) | 243 | 300 | 90 | 80 | 100 | 90 | 80 | 88 |
| | | | 500 | 10 | 0 | 0 | 10 | 20 | 8 |
| | Blood | | 0·5 | 0 | 0 | 50 | 0 | 0 | 10 |
| | (mg/dl) | | 1·0 | 100 | 100 | 50 | 100 | 100 | 90 |
| | Bilirubin | | 0 | 20 | 20 | 30 | 20 | 10 | 20 |
| | (mg/dl) | | 0·2 | 80 | 80 | 70 | 80 | 90 | 80 |
| | Ketones | | 0 | 100 | 100 | 100 | 80 | 100 | 96 |
| | (mg/dl) | | 5 | 0 | 0 | 0 | 20 | 0 | 4 |
| | pH | 8·5 | 8·5 | 30 | 10 | 0 | 0 | 20 | 12 |
| | | | 9·0 | 70 | 90 | 100 | 100 | 80 | 88 |
| | Protein | | 200 | 30 | 10 | 0 | 10 | 80 | 26 |
| | (mg/dl) | 224 | 300 | 70 | 90 | 30 | 90 | 20 | 60 |
| | | | 400 | 0 | 0 | 70 | 0 | 0 | 14 |
| | Glucose (mg/dl) | 1204 | +4 | 100 | 100 | 100 | 100 | 100 | 100 |
| Kova-Trol I | Blood (mg/dl) | | 1·0 | 100 | 100 | 100 | 100 | 100 | 100 |
| | Bilirubin (mg/dl) | | 10 | 100 | 100 | 100 | 100 | 100 | 100 |
| | Ketones | | 45 | 0 | 10 | 40 | 30 | 0 | 16 |
| | (mg/dl) | | 60 | 90 | 90 | 60 | 50 | 80 | 74 |
| | | | 80 | 10 | 0 | 0 | 20 | 20 | 10 |
| | pH | | 8·0 | 0 | 0 | 10 | 0 | 0 | 2 |
| | | 7·5 | 8·5 | 60 | 20 | 90 | 80 | 100 | 70 |
| | | | 9·0 | 40 | 80 | 0 | 20 | 0 | 28 |
| | Protein | | 30 | 0 | 0 | 0 | 20 | 0 | 4 |
| | (mg/dl) | 38 | 50 | 50 | 0 | 50 | 80 | 70 | 50 |
| | | | 70 | 50 | 100 | 50 | 0 | 30 | 46 |
| | Glucose | | 200 | 10 | 0 | 0 | 0 | 0 | 2 |
| | (mg/dl) | 183 | 300 | 90 | 60 | 40 | 90 | 60 | 68 |
| | | | 500 | 0 | 30 | 50 | 10 | 40 | 26 |
| | | | 1000 | 0 | 10 | 10 | 0 | 0 | 4 |
| | Blood | | 0·1 | 0 | 0 | 0 | 20 | 40 | 12 |
| Kova-Trol II | (mg/dl) | | 0·2 | 20 | 0 | 70 | 80 | 60 | 46 |
| | | | 0·5 | 80 | 100 | 30 | 0 | 0 | 42 |
| | Biliburin | | 1·0 | 100 | 30 | 60 | 90 | 100 | 76 |
| | (mg/dl) | | 2·0 | 0 | 70 | 40 | 10 | 0 | 24 |
| | Ketones | | 5 | 10 | 0 | 0 | 0 | 0 | 2 |
| | (mg/dl) | | 10 | 20 | 0 | 0 | 0 | 0 | 4 |
| | | | 20 | 10 | 0 | 0 | 0 | 0 | 2 |
| | | | 30 | 60 | 60 | 0 | 0 | 10 | 26 |
| | | | 45 | 0 | 30 | 0 | 10 | 40 | 16 |
| | | | 60 | 0 | 10 | 70 | 90 | 50 | 44 |
| | | | 80 | 0 | 0 | 30 | 0 | 0 | 6 |

** Instrument display levels.

was probably due to the sensitivity of the test pad being lower for erythrocytes than for free haemoglobin. The Clinitek Auto 2000 provides only qualitative results for haemoglobin content so acceptable ranges were produced by arbitrarily assigning semiquantitative values to qualitative expressions; 92% of results fell into the assigned ranges.

Figures 5 and 6 show the distribution of results obtained with urines artificially supplemented with acetoacetic acid and bilirubin. No false positives for ketone-bodies or bilirubin were reported. The Super Aution Analyzer appears to overestimate, while Urotron RL9 is insufficiently sensitive with a detection limit for ketones of approximately 15 mg/dl (see figure 5). Bilirubin recovery

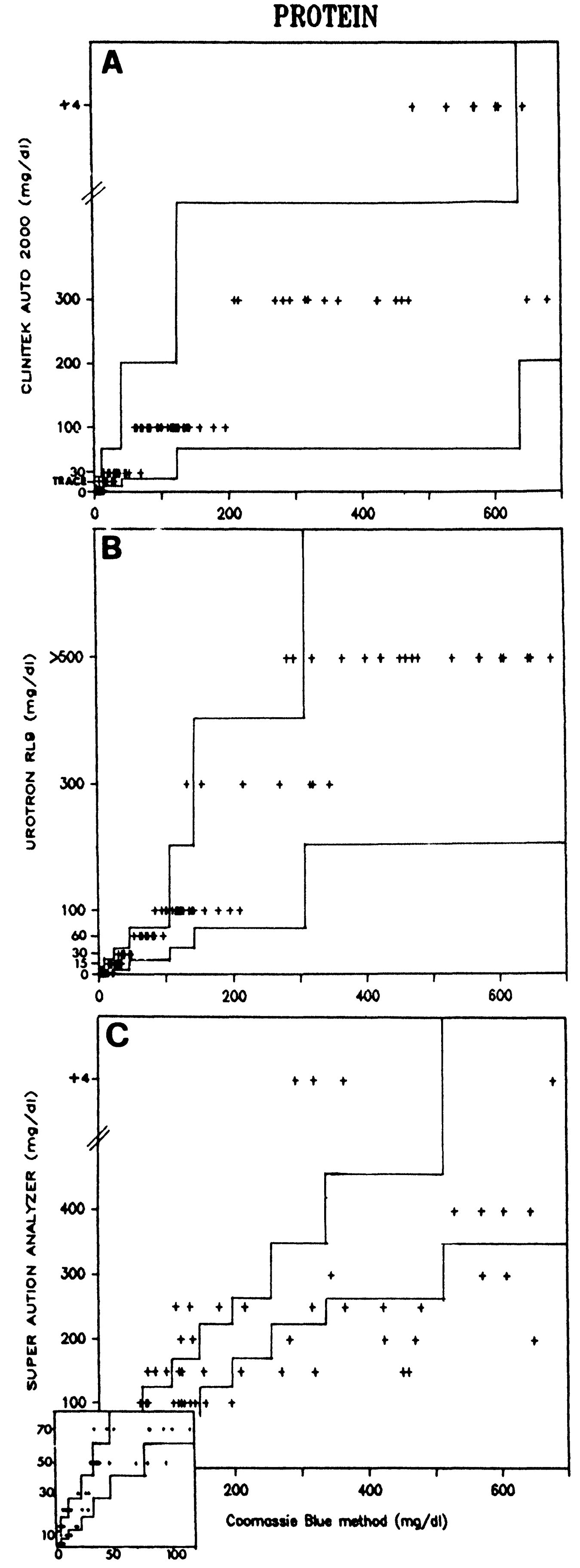

*Figure 1. Protein concentrations in urine samples obtained by the Clinitek Auto 2000 (a), Urotron RL9 (b) and Super Aution Analyzer (c) (y-axis) compared with the Coomassie blue method (x-axis).*

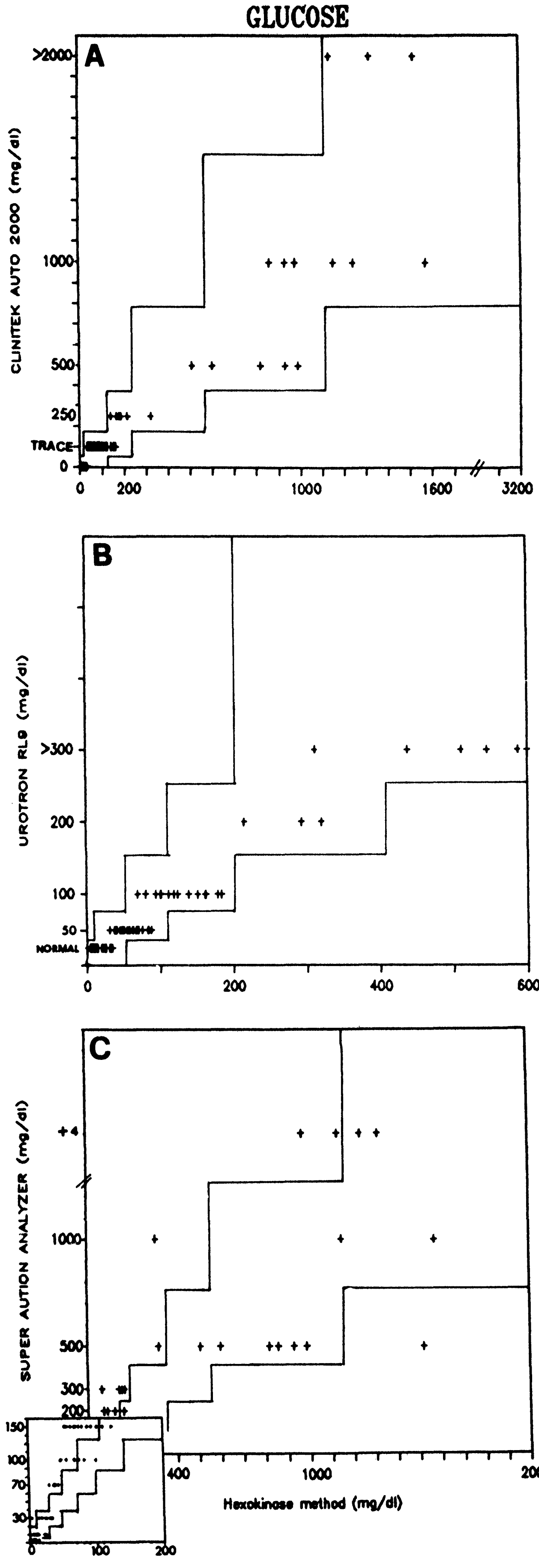

*Figure 2. Glucose concentration in patients' urines obtained by the Clinitek Auto 2000 (a), Urotron RLg (b) and Super Aution Analyzer (c) (y-axis), compared with the hexokinase method (x-axis).*

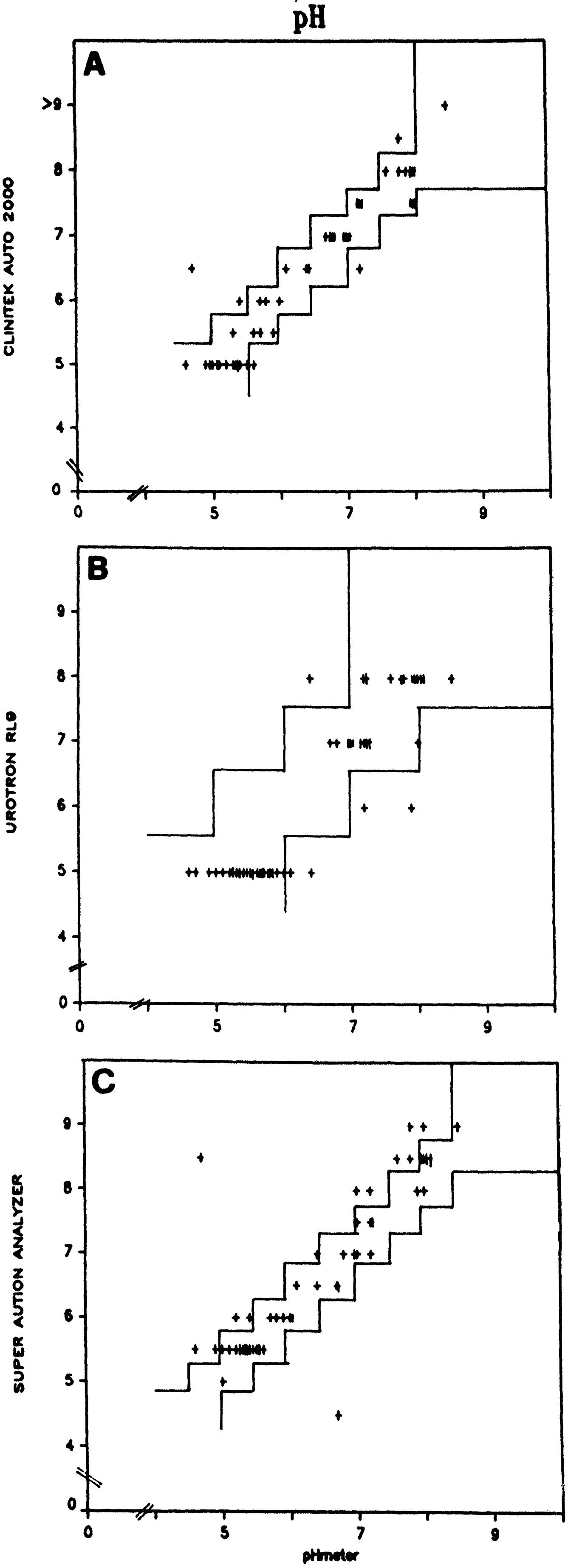

Figure 3. The pH of patients' urines obtained by the Clinitek Auto 2000 (a), Urotron RL9 (b) and Super Aution Analyzer (c) (y-axis), compared with a pHmeter (x-axis).

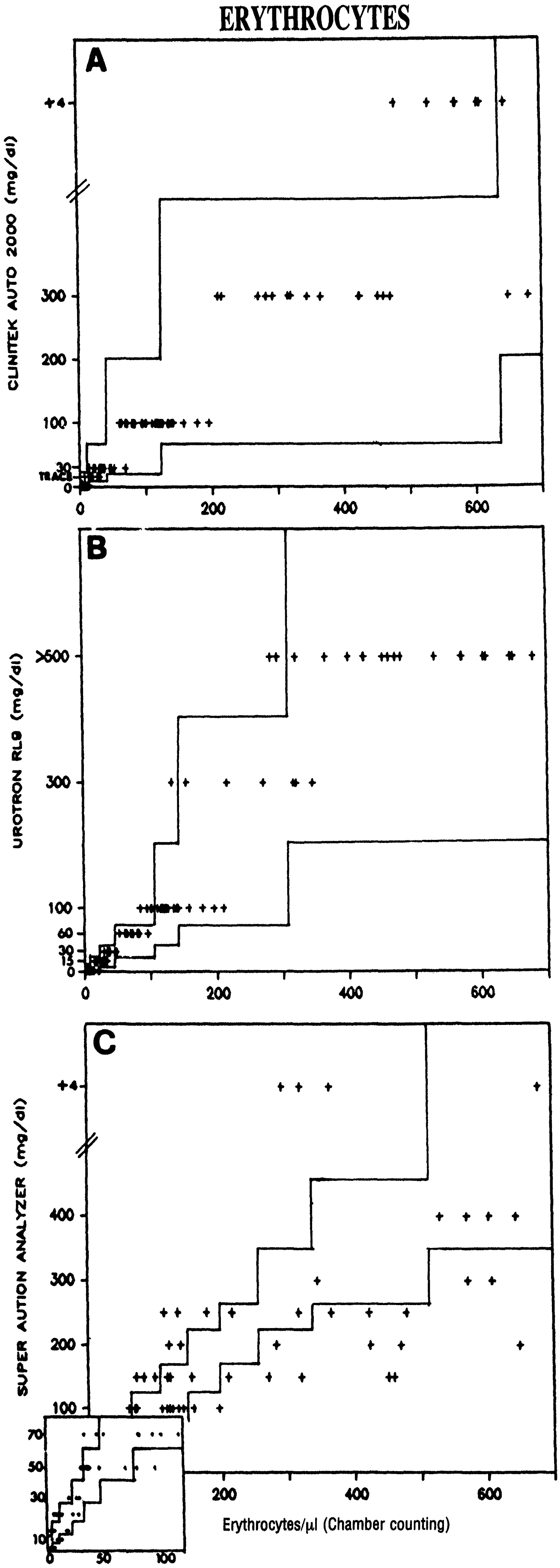

Figure 4. Results for the estimation of haemoglobin in urine samples as made by the Clinitek Auto 2000 (a), Urotron RL9 (b) and Super Aution Analyzer (c) (y-axis), compared with the reference method (chamber counting – x-axis).

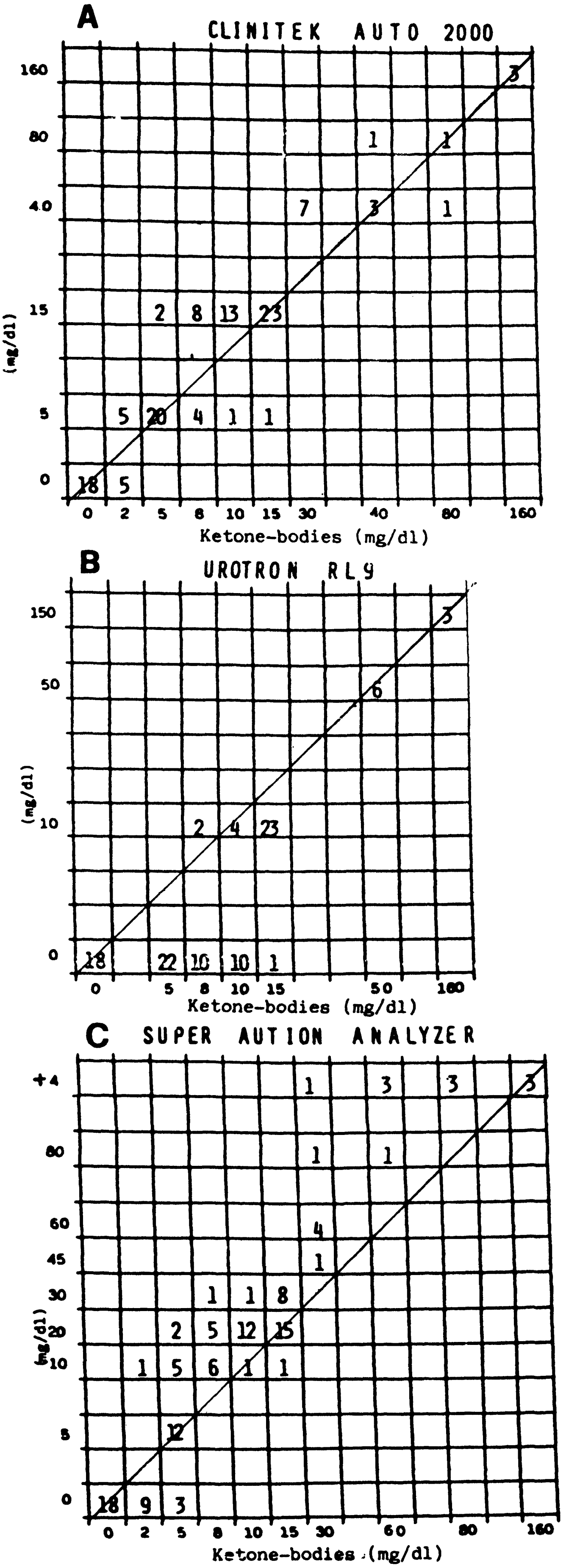

*Figure 5. Recovery experiments for ketone-bodies: (a) Clinitek Auto 2000, (b) Urotron RL9, (c) Super Aution Analyzer.*

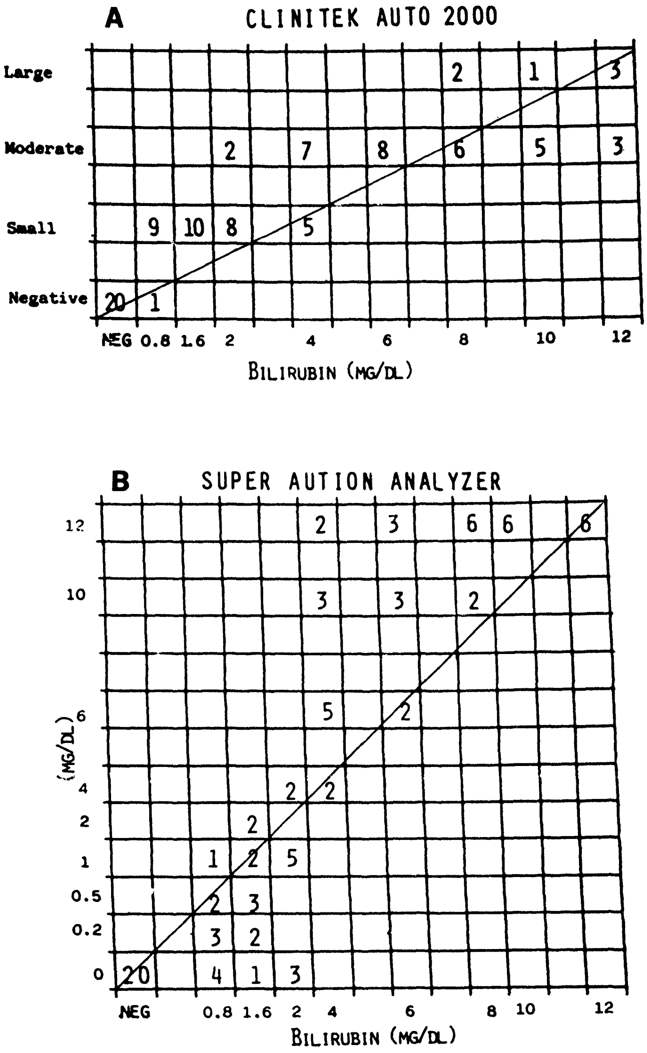

*Figure 6. Recovery experiments for bilirubin: (a) Clinitek Auto 2000, (b) Super Aution Analyzer.*

experiments could not be done with the Urotron RL9; the test strips were completely unreactive to the bilirubin used in the experiments. The results obtained with the other two instruments are shown in figure 6.

*Practicability*

The operation of all the instruments was simple; maintenance required only a few minutes a day and no malfunctions were observed during the evaluation period which lasted about six months. The volume of specimen needed for the three urine-analysers differed. With the Urotron and Super Aution test strip dipping requires at least 12 ml; the minimal volume required for the Clinitek was 2 ml.

### Conclusions

For the mechanization of urinalysis, the evaluated instruments based on reflectance photometry, appeared to be rapid and practical. However, though the precision and accuracy are acceptable, it seems that the optimum is not still achieved. From figures 1–4 and tables 2–4, it will be seen that agreement with the comparison method and precision tends to be better when the concentration ranges are wider. Because of the borderline phenomenon,

*Table 4. Within-day and between-day precision. (Urotron RL9.)*

| | | Ref. | ** | Within day 1 $N=10$ | 2 $N=10$ | 3 $N=10$ | 4 $N=10$ | 5 $N=10$ | Between day $N=5$ |
|---|---|---|---|---|---|---|---|---|---|
| Lyphochek II | pH | 6·5 | 5·0 | 90 | 60 | 90 | 90 | 100 | 86 |
| | | | 6·0 | 10 | 40 | 10 | 10 | 0 | 14 |
| | Protein | 92 | 60 | 50 | 20 | 50 | 50 | 90 | 52 |
| | (mg/dl) | | 100 | 50 | 80 | 50 | 50 | 10 | 48 |
| | Glucose | 243 | 100 | 0 | 0 | 0 | 0 | 10 | 2 |
| | (mg/dl) | | 200 | 100 | 100 | 100 | 100 | 90 | 98 |
| | Blood | | 150 | 0 | 0 | 0 | 70 | 0 | 14 |
| | (Ery/ul) | | 250 | 100 | 100 | 100 | 30 | 100 | 86 |
| | Bilirubin | | Neg | 50 | 100 | 40 | 100 | 60 | 70 |
| | (mg/dl) | | 0·5 | 50 | 0 | 60 | 0 | 40 | 30 |
| | Ketones | | Neg | 100 | 100 | 100 | 100 | 100 | 100 |
| | (mg/dl) | | | | | | | | |
| Kova-Trol I | pH | 8·5 | 8·0 | 70 | 10 | 30 | 80 | 90 | 56 |
| | | | 9·0 | 30 | 90 | 70 | 20 | 10 | 44 |
| | Protein | | 60 | 0 | 0 | 30 | 0 | 0 | 6 |
| | (mg/dl) | 224 | 100 | 0 | 100 | 70 | 0 | 100 | 54 |
| | | | 300 | 50 | 0 | 0 | 90 | 0 | 28 |
| | | | 500 | 50 | 0 | 0 | 10 | 0 | 12 |
| | Glucose | 1204 | 300 | 100 | 100 | 100 | 100 | 100 | 100 |
| | (mg/dl) | | | | | | | | |
| | Blood | | 250 | 100 | 100 | 100 | 100 | 100 | 100 |
| | (Eryl/ul) | | | | | | | | |
| | Bilirubin | | Neg | 0 | 10 | 30 | 0 | 0 | 8 |
| | (mg/dl) | | 0·5 | 0 | 30 | 70 | 0 | 0 | 20 |
| | | | 1·5 | 100 | 60 | 0 | 0 | 100 | 52 |
| | | | 3·0 | 0 | 0 | 0 | 100 | 0 | 20 |
| | Ketones | | 10 | 0 | 10 | 0 | 0 | 10 | 4 |
| | (mg/dl) | | 50 | 100 | 90 | 100 | 100 | 90 | 96 |
| Kova-Trol II | pH | 7·5 | 8·0 | 100 | 100 | 100 | 100 | 100 | 100 |
| | Protein | 38 | 15 | 0 | 20 | 80 | 60 | 80 | 48 |
| | (mg/dl) | | 30 | 100 | 80 | 20 | 40 | 20 | 52 |
| | Glucose | 183 | 100 | 100 | 0 | 0 | 100 | 100 | 60 |
| | (mg/dl) | | 200 | 0 | 100 | 100 | 0 | 0 | 40 |
| | Blood | | 10 | 0 | 0 | 50 | 0 | 0 | 10 |
| | (Eryl/ul) | | 50 | 100 | 100 | 50 | 100 | 100 | 90 |
| | Bilirubin | | Neg | 0 | 60 | 100 | 0 | 30 | 38 |
| | (mg/dl) | | 0·5 | 70 | 40 | 0 | 100 | 70 | 56 |
| | | | 1·5 | 30 | 0 | 0 | 0 | 0 | 6 |
| | Ketones | | Neg | 0 | 0 | 100 | 0 | 30 | 26 |
| | (mg/dl) | | 10 | 100 | 100 | 0 | 100 | 70 | 74 |

** Instrument display levels.

the results falling in adjacent ranges must be accepted [2]. Therefore, when the instrument reports for example, 100 mg/dl of glucose, the real value could be between 60 or 190 mg/dl (figure 2[*b*]). When the number of intervals is increased in an effort to reduce such an uncertainty, as for the Super Aution Analyzer, there appears to be greater inaccuracy. Figures 1–4 show the higher number of unacceptable results with the Super Aution Analyzer due to the greater number of concentration intervals used to give results.

The semiquantitative expression of results appears to be a drawback which should be eliminated to improve the clinical significance of urine analysis, otherwise the urine test with dipsticks can only be a screening test to be verified with more accurate and precise quantitative methods if pathological or discordant reports are obtained.

In conclusion, no major differences appear to exist in the analytical quality of the three analysers; they represent the state of art of urine analysis but an improvement in accuracy is desirable.

### References

1. Guder, W. G. and Heidland, A., *Journal of Clinical Chemistry and Clinical Biochemistry*, **24** (1986), 611.

2. Haeckel, R., Bonini, P. A., Ceriotti, F., Kutter, D. and Vonderschmitt, D. J., *Journal of Clinical Chemistry and Clinical Biochemistry*, **23** (1985), 473.

3. Donohoe, G. A., Geary, T. D. and Jennings, R. D., *IFCC Document No. 4, Evaluation of Instrumentation in Clinical Chemistry* (1982).
4. White, G. H. and Fraser, C. G., *Journal of Automatic Chemistry*, **6** (1984), 122.
5. James, G. P. and Bee, D. E., *Clinical Chemistry*, **25** (1979), 996.
6. Peterson, J. I. and Young, D. S., *Analytical Biochemistry*, **23** (1968), 301.
7. Bradford, M. A., *Analytical Biochemistry*, **72** (1976), 248.
8. Hoeltge, G. A. and Ersts, A., *Pathology*, **73** (1980), 403.
9. Bandi, Z. L., Myers, J. L., Bee, D. E. and James, G. P., *Clinical Chemistry*, **28** (1982), 2110.
10. Smalley, D. L. and Bradley, M. E., *Clinical Chemistry*, **31** (1985), 90.

### ANALYTICON 88

*Olympia Conference Centre, London, 11–13 October 1988*

Highlights of the 1988 Analyticon include:

*Inductively coupled plasmas*

Chemical analysis using inductively coupled plasma (ICP) sources is the subject of a morning session of Analyticon 88 on Tuesday 11 October. This session will contain material of interest both to those who may wish to familiarize themselves with the potential of the ICP for chemical analysis and to those who would like a review of current developments to extend the technique.

The speakers will adopt a tutorial presentation and will be aiming to demonstrate both the developments of ICP-OES, which have already made it a powerful technique for the determination of metals (its inherent advantages as a spectroscopic source, developments in sample preparation methods, and industrial applications of ICP-OES), and the applications of Fourier Transform methods which are capable of achieving theoretical resolution and potentially bringing improvements in both selectivity and signal-to-noise ratio.

*Colin Watson* has convened this session and the speakers will include *Dr C. W. McLeod* from the Centre for ICP Spectrochemical Analysis at Sheffield City Polytechnic, and *Dr E. J. Newman* who is Quality Assurance Manager and Chief Analyst at BDH Ltd.

*Process analysis and control*

A session on this topic is being convened by *R. Mackinson* of the BP Research Centre. This will be held during the morning of 13 October.

*Polymers and electronics*

The Thermal Methods Group of the Royal Society of Chemistry Analytical Division is convening a session of Analyticon 88 entitled 'Polymers and Electronics'. This will be in the morning of Thursday 13 October and will be chaired by *Peter Haines* of the School of Chemical and Physical Sciences at Kingston Polytechnic.

*Atomic absorption spectrometry*

A tutorial style of presentation will be adopted for this session of Analyticon 88 which has been convened by *Colin Watson* for the afternoon of Tuesday 11 October. The emphasis will be on practical applications of atomic absorption spectrometry.

Getting the best from flame atomic absorption will be the topic covered by *Dr Malcolm S. Cresser* from the Department of Soil Science at the University of Aberdeen. *Dr S. J. Haswell* from the School of Chemistry at Thames Polytechnic will consider novel sample preparation and introduction techniques in AAS. A critical appraisal of recent developments in electrochemical AAS will be presented by *Dr D. Littlejohn* from the Department of Pure & Applied Chemistry at the University of Strathclyde.

*Drug and alcohol abuse*

A special session on health care for laboratory workers is being convened by *Dr J. L. Kearns* who is Medical Director at BUPA Occupational Health. At the time of going to press we have no details of this session but it is expected that it will include discussions on the rising problem of drug and alcohol abuse, as well as general health-care screening for laboratories. This session of Analyticon 88 will be in the afternoon of Tuesday 11 October.

*Expert systems*

*Professor D. L. Massart* who heads the Pharmaceutical Institute at the Free University in Brussels is organizing a seminar session on expert systems to be held in the afternoon of Tuesday 11 October at Analyticon 88. He will be joined by *Dr Buydens* also from the Pharmaceutical Institute laboratory.

*All enquiries regarding Analyticon 88 should be addressed to Scientific Symposia Ltd, 33–35 Bowling Green Lane, London EC1R 0DA. Tel.: 01 837 1212.*

